# Impact of Purchase Preference, Perceived Value, and Marketing Mix on Purchase Intention and Willingness to Pay for Pork

**DOI:** 10.3390/foods10102396

**Published:** 2021-10-09

**Authors:** Mei-Ling Kung, Jiun-Hao Wang, Chaoyun Liang

**Affiliations:** Department of Bio-Industry Communication and Development, National Taiwan University, Taipei 10617, Taiwan; d05630003@ntu.edu.tw (M.-L.K.); wangjh@ntu.edu.tw (J.-H.W.)

**Keywords:** marketing mix, perceived value, pork, purchase intention, purchase preference, willingness to pay

## Abstract

This study explored the effects of purchase preference, perceived value, and marketing mix on consumers’ purchase intention and willingness to pay for pork in Taiwan. A questionnaire was distributed to pork consumers in an online platform, and a total of 1042 valid samples were collected. An analysis of the questionnaire responses revealed three purchase preference factors, namely flavour, certification marks, and added features; four perceived value factors, namely functional, social, conditional, and emotional value; and four marketing mix factors, namely promotion, convenience, product, and price marketing. Functional value, purchase frequency, conditional value, and product marketing positively affected purchase intention. Promotional marketing, monthly disposable income, and social value were the main positive factors in increased willingness to pay. Consumers who were men, had a lower educational level, purchased large quantities of pork at one time, or frequently purchased pork exhibited higher purchase intention than did other consumers. Those who were men, had higher educational attainment, had a higher monthly disposable income, or held a management position were more willing to purchase pork at a premium price.

## 1. Introduction

Pork is an excellent source of nutrition and is the most consumed type of meat globally. Consumers exhibit various preferences for internal and external cues related to meat products and purchase different combinations of pork products on the basis of these preferences [1,2,3]. Perceived value affects consumers’ decisions to acquire new experiences, and the extent to which this effect is associated with willingness to pay (WTP) is based on their consumption behaviour [4]. Consumers obtain information regarding pork products through a variety of promotional activities, which affect their impressions of the products, their purchase decisions, and their WTP [5,6]. However, little market research has been conducted that integrates purchase preference, perceived value, and supplier marketing strategies.

More than 50% of pork worldwide is produced and consumed in Asia [7]. Most studies on pork consumption have focused on Europe, Africa, and the United States [8,9,10,11], with research on pork consumption in Asia lacking. Factors that affect consumers’ behaviour and preferences vary depending on cultural background and local customs [12,13]. Studies have revealed that consumers’ socioeconomic and demographic characteristics also affect intention to purchase pork and WTP [1,5,14,15].

After the outbreak of aphthous fever in Taiwan in 1997, Japan immediately banned pork imports from Taiwan. At the time, pork exported from Taiwan accounted for 40% of Japan’s total pork imports [16]. The World Organization for Animal Health (OIE) declared Taiwan free of aphthous fever in June 2020. Shortly thereafter, in 2021, the Taiwanese government approved imports of frozen pork containing ractopamine (an animal feed additive banned in various countries) from the United States. Pork consumption has not been studied since the OIE declared Taiwan an aphthous fever–free country. These events led to uncertainty in the industry and related markets and generated an urgent need to update the understanding of the pork market.

This study explored the following topics: (1) the preferences and evaluation processes of pork consumers in Taiwan; (2) the marketing mix strategies of which they approve; (3) how purchase preference, perceived value, and marketing mix affect consumer purchase intention and WTP; and (4) which demographic variables are associated with purchase intention and WTP. This study can help pork suppliers gain further understanding of consumer psychological and behavioural trends in Taiwan and thus enable producers and retailers to devise precise strategies that meet consumer needs. The results can also serve as a reference for international trading companies evaluating import and export markets, thereby promoting the sustainable management of the pork industry.

## 2. Literature Review

### 2.1. Pork Purchase Intention, WTP, and Consumer Characteristics

Consumers spend more on pork than on any other agricultural product in Taiwan. As of 2019, an average Taiwanese person consumes approximately 36.84 kg of pork annually, most of which is produced domestically. Taiwan ranks among the top 10 countries in pork consumption worldwide [17]. Global pork consumption continues to increase, and the United States has become the largest exporter of pork. U.S. pork accounts for 30% of global pork consumption [18]. In Europe, pork accounts for 47% of total meat production, and this proportion continues to increase. In 2021, global pork production is expected to increase by 2% from 2020, reaching 103.8 million tons. China is the largest pork production country, accounting for 40% of global production; it also consumes the most pork [19].

Purchase intention is defined as a tendency to purchase a product or service [20]. It is expressed by phrases such as ‘I prioritise buying this product over other products’, and ‘I buy this product often’ [21,22]. Marketing studies have used consumption frequency to evaluate purchase intention [12,23]. High purchase intention indicates a high likelihood that a consumer will buy a certain product [24].

WTP denotes the maximum price that a consumer is willing to pay for a product [25]. It is often used to determine product characteristics that are prioritised by consumers [9]. WTP is highly correlated with product price [26]. In Taiwan, pork price is determined through auctions and fluctuates daily with supply and demand. Consequently, it cannot be estimated precisely. To address this problem, this study used the proportion of consumers who are willing to pay a premium [9,26] as the indicator of WTP. Studies have verified that WTP determined using a scale evaluating willingness to pay a premium is consistent with that determined using other standard methods commonly used by economists [27].

Pork consumption varies between consumers from different/of/with regional environments, social culture, daily life experiences, and eating habits [5,14]. Consumers in different countries react differently towards product certification marks, which in turn affects their consumption behaviour [1]. Gender, educational level, and purchase location affect pork purchase intention [12]. Purchase intention towards new food products increases with educational level [28]. Consumer age and income affect preferences and WTP [29]. Gender and socioeconomic status affect behaviour when purchasing safe food, and men are more willing to purchase food without safety certifications than are women [15]. WTP varies between different regions [30]. Therefore, where consumers live is a crucial factor affecting their WTP for pork products.

### 2.2. Effect of Purchase Preference on Purchase Intention and WTP

Consumers have distinct preferences for various product characteristics. Limited by their budgets, consumers purchase various combinations of products on the basis of their preferences [3]. Pork purchase preference is related to sensory pleasure, visual appearance, and flavour [5], which affect perception and emotion, causing consumers to maintain the same preferences [31]. When evaluating product quality or functionality, consumers often consider internal and external cues related to product characteristics [2,3]. The internal cues of meat products include taste and freshness, and external cues include the appearance, smell, country of origin, price, safety and quality certification, method of production, and trust in the supplier [1,32].

The product characteristics of pork include nutritional value (e.g., cholesterol, calories, protein, and fat content), quality (e.g., freshness and tenderness), safety (e.g., pathogen testing, absence of antibiotics, and absence of artificial additives), and convenience (ease of purchase and preparation) [33]. The appearance, flavour, country of origin, and production information of pork affect consumer selection [10]. When buying pork, consumers prioritize flavour (75.6%), followed by preparation convenience (e.g., a variety of recipes, 30.7%), price (23.9%), usability (23.3%), and nutritional value (16.7%) [34]. Other scholars have indicated that consumer WTP is affected by preference and the quality certification, traceability, country of origin labelling, and appearance of pork [29]. Consumer WTP for pork is affected by four factors: government certification, country of origin, price, and an antibiotic residue–free marking. Pork purchase volume decreases with increasing price, and WTP decreases with increasing purchase volume [15].

### 2.3. Effect of Perceived Value on Purchase Intention and WTP

Perceived value denotes consumers’ overall evaluation of a product or service [35]. It provides a sense of novelty, elicits curiosity, and fulfils the need to acquire knowledge. Perceived value is a crucial factor affecting consumers who are deciding on whether to acquire a new experience [4]. Sheth, Newman, and Gross categorised customer value into functional, emotional, social, and conditional value [36]. The four values, which are independent, affect consumer decisions, and the extent of their effects is based on actual consumption situations [4].

Functional value is associated with the benefits or effects gained by consumers when using a product [37,38]. Common functional values include product quality, reliability, durability, and price [39]. Pork is rich in nutrition and an excellent source of protein, iron, and zinc. It also contains abundant thiamine, fat, and carbohydrates. In addition, pig livers and kidneys are rich in Vitamin A, Vitamin B, and folate. The availability of information regarding the nutritional value of pork increases purchase intention [40,41]. Moreover, satiety, logical pricing, and value for money are factors prompting consumers to purchase meat products [37,40].

Emotional value is associated with psychological needs and emotional states [38]. This type of value reflects a product’s ability to elicit emotions and interest in users. Emotional value is a social psychological factor [37] essential to predicting purchase intention [4]. Scholars have identified satiety and happiness gained from consuming meat as the emotional value of meat products [33,40]. Moral concerns such as guilt reduce consumer intention to purchase meat [42]. Emotional value is also affected by way of life and cultural background. For example, consumers who are Muslim are prohibited from eating pork because it is considered unclean [13].

Social value refers to product characteristics that can enhance the personal image or social approval of consumers and associate consumers with specific social [37,39]. Social value also refers to the social effects of a consumption behaviour. These effects include social status and reputation [38]. When consumers use a product to construct and show their status, they distinguish themselves from others and experience a sense of achievement [43]. High social value increases consumer satisfaction with a product [44]. Scholars have argued that essential scale items for evaluating social value explore whether products can enhance a sense of belonging to a group or how others perceive the use or procurement of a product (i.e., whether a product improves the image of consumers or enables them to gain a sense of superiority) [4,45].

Conditional value refers to a product’s ability to provide temporary functions or social value in specific scenarios or in brief, emergency events [36]. This type of value is generated from the moderating effect of a scenario on the relationship between functional value and the perception of an outcome. A scenario can reduce the effect of emotional value on an outcome [4]. Consumer perceptions of a product’s place of origin affect their preference and trust in the product as well as its image. Indeed, place of origin exerts a greater effect on purchase behaviour than does quality. For example, interviewees in a study by Papanagiotou et al. [12] admitted that they preferred pork produced in Greece not because of its quality but because of factors such as social approval, ethnocentrism, emotional displacement, or support for local meat suppliers.

### 2.4. Effect of Marketing Mix on Purchase Intention and WTP

The concept of marketing mix refers to the effective integration of marketing strategies and activities during product sales [46]. In general, marketing mix consists of the “4Ps”, namely promotion, place, product, and price [46,47]. Consumers receive information on meat products through brand advertisements and promotional activities, which in turn affect their impression and expectation of the products as well as their choices, purchase decisions, and WTP [5,6]. Different marketing mix strategies can be employed together to generate a synergistic effect [48]. In addition, marketing should involve consideration of individual consumer perceptions [11]. This study referred to Chen’s study to design a marketing mix scale [47].

Promotion refers to activities that are conducive to product marketing and increasing product sales [47]. It is used to showcase a product to consumers and encourage them to purchase it [49]. Promotional media include television and radio broadcasts, pamphlets, social network sites, advertisements, exhibitions, and instore shopping guides [48]. Consumers in Western countries trust scientists and food suppliers more than they do governments [50], whereas consumers in Eastern countries consider governments, scientists, research institutions, and physicians to be equally reliable sources of information [51]. Most consumers are willing to pay a premium for food products that comply with the safety standards stipulated by a government or those offered by a famous brand [52]. However, consumers’ trust in brands and certification agencies varies between countries. In addition, consumer intention to purchase a product is affected by the attractiveness of a celebrity who endorses it [53].

Place refers to a location where consumers can purchase a product in a convenient manner [54]. It is also a crucial factor in consumer selection of meat products [23]. Studies have indicated that consumers prefer processed meat products because of their preparation convenience [8,11]. In addition, the packaging, preservation, market type, and sales environment of pork products are closely related to the international trade of these products [55]. In addition, Ortiz et al. [56] discovered that compared with younger consumers, older consumers are less willing to buy vacuum-packaged food.

Product refers to a commodity or service provided by a vendor [49]. A product provides benefits and value required by consumers, which include its characteristics, packaging, labelling, and added value [54]. When consumers evaluate a food product, they consider factors such as safety standards, product type or differentiation, packaging, diverse functions, ingredient labelling, and brand [48]. In general, consumers hold a positive view of pork products and their quality, and most consumers consider pork a healthy and convenient product worthy of its cost [57]. Some countries require all meat products to have county-of-origin labelling because this information is considered essential for food retailers, supply chains, and consumers. Food safety marks and brand labelling provide crucial information on food suppliers and products, allowing consumers to determine product safety and quality [58].

Price refers to the amount of money that consumers pay for a product or service [49]. Pork, like most agricultural products, is a common and highly accessible food product, and consumers are willing to purchase it as long as the price is reasonable [41]. When consumers cannot determine the quality of meat, they use its price to make a judgement [12]. Some consumers are sceptical about the quality of cheap pork; some believe that cheap pork can still be of excellent quality; and some posit that expensive pork has superior quality [59]. Therefore, price is a quality indicator that affects consumer choice of pork products, and pricing becomes a marketing strategy. Each consumer perceives price rationality differently, but their perception is often affected by the price of other or competing products [48].

## 3. Methods

Purposive sampling was adopted to select the respondents of an online questionnaire survey. To ensure sampling adequacy, the introduction to the questionnaire stated that the survey was intended for consumers who bought pork. A website link to the questionnaire was shared through pig farming associations and networks in Taiwan as well as pork vendors and their customer databases to collect data for analysis. The questionnaire comprised six sections, namely purchase preference, perceived value, marketing mix, purchase intention, WTP, and demographic variables. The variables and their items are listed in the Appendix A.

The content validity of the questionnaire items was verified by five experts, including a professor of husbandry, a meat product expert, and a government official in the agriculture and husbandry industry. Subsequently, over 10 pork consumers were invited to verify the face validity, or readability, of the items. The questionnaire was revised on the basis of their suggestions, and the items were scored using a 6-point rating scale from 1 (strongly disagree) to 6 (strongly agree). All the items were to be answered, avoiding the problem of missing data. Reponses collected from a pretest were analysed to verify the validity and reliability of the items.

The questionnaire was distributed using the SurveyCake platform in October and November of 2020. To ensure compliance with research ethics, the first page of the questionnaire clearly stated the research objective, assured the respondents that the responses were collected anonymously, and explained that all statistics were to be presented as mean scores, thus encouraging respondents to answer all items without concern for their identities being revealed. The collected data were analysed using SPSS for Windows 21.00. Demographic variables were examined using descriptive statistics. Factor analysis, multiple regression, *t* testing, and a one-way analysis of variance (ANOVA) were performed to address the research topics.

## 4. Results

### 4.1. Descriptive Statistics

A total of 1112 completed questionnaires were collected. Samples with a response variance of 0 or straight-lined responses were excluded, and 1042 valid samples were retained (Table 1).

### 4.2. Factor Analysis for Research Questions 1 and 2

To address research question 1 (the preferences and evaluation processes of pork consumers in Taiwan) and research question 2 (the marketing mix strategies of which they approve), exploratory factor analysis (EFA) was performed using principal component analysis with varimax rotation to reduce the number of variables for subsequent tests. In our EFA, factors with an eigenvalue of >1 and a factor loading of >0.4 were retained.

The Kaiser–Meyer–Olkin (KMO) value of the purchase preference scale was 0.89, and the Bartlett sphericity test result was significant (*p* = 0.00, *χ*^2^ = 6351.94, *df* = 153), indicating that the scale was suitable for factor analysis. After the analysis, three preference factors were extracted, namely flavour (8 items), certification marks (6 items), and added feature (2 items). Four items were removed from the EFA model. The total reliability (α values) of this scale was 0.82, and the total variance explained was 54.02%, indicating favourable reliability and validity.

The KMO value of the perceived value scale was 0.81, and the Bartlett sphericity test result was significant (*p* = 0.00, *χ*^2^ = 7706.55, *df* = 231), indicating the suitability of factor analysis. The four factors extracted were functional value (9 items), social value (6 items), conditional value (5 items), and emotional value (2 items). Three items were removed from the EFA model. The total reliability (α values) of this scale was 0.82, and the total variance explained was 50.76%, indicating favourable reliability and validity.

The KMO value of the marketing mix scale was 0.880, and the Bartlett sphericity test result was significant (*p* = 0.00, *χ*^2^ = 9497.47, *df* = 231), indicating the suitability of factor analysis. Four factors were extracted from the scale, namely promotion (8 items), convenience (6 items), product (2 items), and price (3 items). Three items were removed from the EFA model. The total reliability (α values) of this scale was 0.85, and the total variance explained was 56.33%, indicating favourable reliability and validity.

The total reliability (α values) of the purchase intention scale (3 items) was 0.91, indicating high reliability. The total variance explained was 85.35%, indicating high validity.

A Pearson’s correlation analysis indicated that each correlation coefficient between the variables was lower than 0.6 (Table 2), indicating good discriminant validity.

### 4.3. Regression Analysis for Research Question 3

To address research question 3 (how purchase preference, perceived value, and marketing mix affect consumer purchase intention and WTP), regression analysis was conducted. The model was determined to be significant (*p* < 0.05) and have an explanatory power (*R*^2^) of 0.43 (Table 3). Functional value exerted the greatest positive effect on purchase intention, followed by purchase frequency, conditional value, and product marketing. Emotional value exerted a significant negative effect on purchase intention. The other factors affected purchase intention nonsignificantly.

This study also analysed the effects of purchase preference, perceived value, marketing mix, and purchase frequency on WTP for pork products. The constructed model was determined to be significant (*p* < 0.001) and exhibited an explanatory power (*R*^2^) of 0.19 (Table 4). The results demonstrated that promotional marketing and monthly disposable income were the strongest positive factors affecting WTP, and that social value was a positive factor of WTP. Price marketing was the strongest negative factor, followed by product marketing, functional value, and emotional value. The remaining factors affected WTP nonsignificantly.

### 4.4. Difference Analysis for Research Question 4

To address research question 4 (which demographic variables are associated with purchase intention and WTP), an independent sample *t* test and one-way ANOVA (with a Scheffé post hoc test) were performed. The results of the one-way ANOVA revealed that purchase intention and WTP differed nonsignificantly among consumers of different ages and places of residence.

#### 4.4.1. Gender Differences

The independent samples *t* test revealed that purchase intention and WTP differed significantly between genders. Specifically, men demonstrated higher purchase intention and WTP than did women (Table 5).

#### 4.4.2. Educational Level Differences

The ANOVA results revealed that purchase intention and WTP differed significantly among consumers with different educational levels (Table 6). Specifically, consumers with a senior high school or vocational school diploma or below exhibited higher purchase intention than did those who with a junior college or university diploma. However, WTP increased with educational attainment.

#### 4.4.3. Disposable Income Level Differences

Purchase intention differed nonsignificantly among consumers with different monthly disposable income levels, whereas WTP increased with monthly disposable income level (Table 7). Specifically, WTP increased with monthly disposable income level.

#### 4.4.4. Product Type Differences

Consumers’ purchase intention differed nonsignificantly among pork product type, and consumers’ WTP differed significantly among pork product type (Table 8). The Scheffé test revealed that consumers who frequently purchased frozen or chilled pork exhibited higher WTP.

#### 4.4.5. Purchase Frequency Differences

The ANOVA results revealed that purchase intention differed significantly with purchase frequency, whereas WTP differed nonsignificantly. The Scheffé test results revealed that consumers with high purchase frequency exhibited higher purchase intention than did those with low purchase frequency (Table 9).

## 5. Discussion

### 5.1. Effect of Purchase Preference, Perceived Value, and Marketing Mix on Purchase Intention

The regression analysis revealed that functional value, purchase frequency, conditional value, and product marketing significantly affected purchase intention. Specifically, functional value exerted the greatest effect, indicating that nutrition, fun, and money are essential to purchase intention. The strong influence of purchase frequency indicates the crucial role of customer relationship management. The effects of conditional value reflect consumers’ belief in information regarding the texture and characteristics of the pork products, cooking methods or recipes for different types of pork, and support for domestic farmers. The influence of product marketing represents pork consumers’ emphasis on freshness and safety certification. The results of this study concur with those of multiple international studies [12,32,40,60].

Emotional value reduced purchase intention if consumers felt a sense of guilt from eating pork because of emotional factors such as environmental awareness, support for vegetarianism, religious beliefs, and moral opposition to slaughter or because they perceived pig farms as being dirty. This result is consistent with those of studies elsewhere [61,62,63].

Purchase preference, social value, promotion marketing, and convenience marketing did not exert a significant effect on purchase intention. In Taiwan, pork is considered an affordable everyday food. Consumers may not be particularly encouraged to purchase pork products by flavour or added features. Consumers usually do not feel a sense of superiority by purchasing pork; consequently, the social value of pork does not affect purchase intention. In addition, the per capita income and consumption power of Taiwan are high, and the price of pork in Taiwan is stable; hence, promotion marketing does not affect purchase intention. Moreover, Taiwan is geographically small and has a high population density, and its highly developed delivery industry enables consumers to easily acquire packaged pork; therefore, convenience marketing is not a factor affecting purchase intention.

### 5.2. Effect of Purchase Preference, Perceived Value, and Marketing Mix on WTP

Promotional marketing, monthly disposable income level, and social value significantly increased WTP for pork. WTP can be increased by promotional marketing that involves endorsements by connoisseurs and executive chefs and professional services provided by vendors in shopping premises. Specifically, WTP increased with disposable income level because individuals with high income levels are capable of purchasing high-quality food at high prices [64]. Products that provide social value, which increases a sense of superiority, can also increase WTP [43]. Regarding conditional value, consumer satisfaction can be improved by providing carefully designed scenarios that enable consumers to acquire new knowledge or added services, which in turn increases their WTP [65]. Overall, suppliers who want to effectively increase WTP should focus on consumers with favourable financial status and devise precise promotional strategies to increase the value of pork products by meeting the psychological needs of such consumers.

Price marketing, product marketing, functional value, and emotional value reduced WTP. In general, consumers are willing to increase the amount they pay for product when they notice a uniqueness or added services. In this study, price marketing emphasised the low price of pork; however, this marketing strategy diminished the perceived value of pork [66], thereby lowering WTP. Product marketing in this study was focused on value for money and reasonable pricing. However, various products exist that can serve as an alternative to pork and meet consumer needs for happiness, satisfaction, and nutrition. Product marketing and functional value related to basic needs did not increase WTP. In addition, emotional value reduced WTP because of consumer support for environmental protection, vegetarianism, and religious beliefs.

Purchase preference, conditional value, and convenience marketing were nonsignificantly correlated with WTP. Pig farming systems differ among countries, and factors such as the dining environment, cooking methods, and personal preferences affect the preference for pork [65]. Because of the wide variety in the factors that affect purchase preference and conditional value, they do not influence WTP. In addition, because numerous places in Taiwan sell pork products that have been presliced and prepackaged, consumers view product convenience, an aspect of convenience marketing, as a basic characteristic of pork. Consequently, convenience marketing does not affect WTP.

### 5.3. Effect of Demographic Variables on Purchase Intention and WTP

Consumers’ purchase intention differed among genders, educational levels, and purchase frequency. Meat consumption reflects social status, which affects male consumers’ purchase of meat products [67]. In this study, men exhibited greater pork purchase intention than did women. Consumers with low educational levels exhibited greater purchase intention; this might be attributable to more highly educated consumers wishing to control their meat consumption [68]. Consumers’ purchase intention did not vary among age groups, place of residence, monthly disposable income level, or type of most frequently purchased pork product. This finding differs from those of previous studies [69,70]. This difference might be attributable to the affordability of pork in Taiwan, where the price of pork is stable and various food products exist as alternatives to pork. Consequently, these factors did not affect purchase intention.

Regarding WTP for pork, men were more willing to pay a premium than were women, and WTP increased with educational attainment, supporting the findings of previous studies [71]. Moreover, consumers with a high monthly disposable income exhibited higher WTP than did those with a low monthly disposable income. This finding is consistent with studies indicating that individuals with high incomes are less sensitive to price. In this study, consumers were willing to pay higher prices for frozen, vacuum-sealed pork imported from other countries or those sold by famous brands. This finding concurs with those of previous studies [71]. WTP differed nonsignificantly between consumers of different ages, places of residence, and purchase frequency. Consumers living in rural areas had larger budgets for purchasing pork than did those living in cities [72]. However, WTP was observed to differ nonsignificantly by place of residence in the present study. Therefore, the difference between the WTP of residents of rural and urban areas requires further research. Finally, consumers who purchased pork once per month exhibited the highest WTP, indicating that individuals who occasionally purchase pork are willing to increase the amount they pay for it.

### 5.4. Research Limitations and Future Inquiries

This study has at least two limitations. (1) This study focused on market surveying; other parameters potentially affecting customers’ purchase intentions and WTP for pork products, such as pork suppliers’ managerial ability and costs, were not examined. (2) The survey was conducted between October and November 2020, during which time the OIE declared Taiwan free of aphthous fever. In the following year, the Taiwanese government lifted the restriction on importing U.S. pork containing ractopamine. These events led to uncertainty in the industry and in market information and in turn might have affected the accuracy of the survey. Despite the aforementioned limitations, the effects of which were minimized, this study is crucial for understanding purchase intentions and WTP for pork products in Taiwan. This investigation provides insight into an integrative approach for the understudied but essential topics of purchase preference, perceived value, and marketing mix.

To address the aforesaid limitations, subsequent research can focus on the following major directions. (1) Focus group interviews can be conducted with consumers or hypermarket managers to collect qualitative data and reinforce the discourse on the causal relationships of relevant variables. (2) The questionnaire used in this study can be further revised. The marketing mix scale can be expanded on the basis of different theories (e.g., the 7P marketing mix model).

## 6. Conclusions and Suggestions

This study examined pork consumers and identified three purchase preference factors, namely flavour, certification marks, and added features; four perceived value factors, namely functional, social, conditional, and emotional value; and four marketing mix factors, namely promotion, convenience, product, and price marketing. Functional value, purchase frequency, conditional value, and product marketing positively affected purchase intention, whereas emotional value negatively affected purchase intention. Promotional marketing, monthly disposable income level, and social value were the main positive factors that increased WTP, whereas price marketing, product marketing, functional value, and emotional value were negative factors that decreased WTP. Consumers who were men, had low educational levels, purchased a large quantity of pork at one time, and purchased pork frequently exhibited higher purchase intention. Consumers who were men, had high educational attainment, had a high monthly disposable income, and held a management job title were more willing to pay a premium for pork.

The study makes academic contributions in five areas. (1) The researchers structurally classified factors related to pork purchase preference, thus filling a gap in the research. (2) Scholars have rarely performed integrative surveys on purchase preference, perceived value, and marketing mix for pork products. This study constructed a fundamental preference–value–marketing framework for monitoring yearly changes in market trends. (3) The results can serve as a reference for the husbandry industry in Taiwan to identify key factors affecting the market, helping suppliers devise plans for industrial development. The results also provide practical implications that facilitate the formulation of strategies for targeting high-income consumers and importing products. (4) The survey instrument and theoretical model developed in this study can be adopted to compare consumers across different countries and reveal consumption and market trends in different regions. This can enable suppliers to evaluate the substitution and competitiveness of pork products in a market, providing a market entry reference for international traders. (5) The results can serve as a reference for authorities in Taiwan to formulate policies for pork exports and subsidies for pork suppliers.

Regarding practical contributions, the results can help pork suppliers and retailers devise more precise marketing strategies and improve their competitiveness and profitability. Pork is a common ingredient in Taiwan. Pork products that emphasise taste, nutritional value, and preparation convenience can attract consumers. To enhance purchase intention through marketing strategies, endorsers can be hired to advertise the juiciness and tenderness of pork products and convey customers’ satisfaction with the products. To improve international competitiveness, the Taiwanese government can reference carcass grading systems in Japan or the United States to devise its own standards for grading the meat and marbling quality of pork, thereby meeting the needs of consumers in different markets. The results indicated that educational attainment and financial status change cognition and behaviour. The increasing public awareness of cold chain technology and food safety and the prevalence of special varieties of frozen pork products imported from abroad or sold by famous domestic brands have increased the Taiwanese population’s acceptance of frozen products packaged in vacuum-sealed bags; even consumers with lower income levels have begun to accept such products. This phenomenon reflects the importance of establishing customer service systems and managing regular customers. The purchase records and personal information of customers can be examined to create suitable promotion strategies and increase repurchase behaviour.

The global market value of pork is large, as is the demand for it. This increases pork export opportunities for Taiwan, particularly since it has been declared free of aphthous fever. Agricultural authorities should constantly monitor changes in domestic and international markets to determine the positioning and value of pork products, thereby helping pork suppliers create marketing plans and analyse competitive advantages conducive to supply chain development [73]. The production end of the Taiwanese pork industry should improve its disease prevention measures to eliminate specific pathogens, thereby reducing export barriers other than duties. The industry should also devise plans for cultivating varieties with special flavour and establishing brands and industrial chains that comply with the Hazard Analysis and Critical Control Point system. This facilitates increasing domestic WTP for pork, shaping the image of Taiwan, and creating international competitive advantages. In Taiwan, retailers can place ractopamine-free marks on U.S.-imported products on their own, and inspection for ractopamine content in pork is loose, causing concerns among consumers. Therefore, pork suppliers should consider consumers’ emphasis on food safety and utilise their preference and support for pork from local farms. By comprehensively controlling each component of the supply chain, managing customer relations, and employing adaptive promotion strategies, the Taiwanese pork industry can produce with value and price flexibility.

## Figures and Tables

**Table 1:** Descriptive statistics of demographic variables (*n* = 1042).

Demographic Variable	Percentage (*n*)							
Gender	Male				Female			
	47.7% (497)				52.3% (545)			
Age	≤45 years			46–55 years		≥56 years		
	32.7% (341)			36.5% (380)		30.8% (321)		
Educational attainment	Senior high school or vocational school or less			Junior college or university		Graduate degree		
	15.5% (162)			52.4% (546)		32.1% (334)		
Place of residence	Northern Taiwan		Central Taiwan		Southern Taiwan		Eastern Taiwan	
	50.3% (524)		15.0% (156)		8.9% (93)		25.7% (268)	
Mean monthly disposable income (NTD)	≤10,000		10,001–30,000		30,001–50,000		≥50,001	
	16.4% (171)		34.5% (359)		21.8% (228)		27.3% (284)	
Most frequently purchased pork product type	Frozen		Chilled		Fresh		Processed	
	9.1% (95)		33.1% (345)		54.5% (568)		3.3% (34)	
Purchase frequency	Daily	Once per 2–3 days		Once per week		Once per month		Rarely
	1.0% (10)	20.3% (212)		48.5% (505)		13.1% (137)		17.1% (178)

**Table 2:** Pearson’s correlation among the variables (*n* = 1042).

	1	2	3	4	5
Purchase preference (1)	1.00				
Perceived value (2)	0.11 ***	1.00			
Marketing mix (3)	0.00	0.49 ***	1.00		
Purchase intention (4)	0.14 ***	0.52 ***	0.32 ***	1.00	
WTP (5)	−0.03	0.16 ***	0.07	0.07 *	1.00

Note: * *p* < 0.05, *** *p* < 0.001.

**Table 3:** Regression analysis of purchase intention (*n* = 1042).

Variable	Purchase Intention				
	Nonstandardised Beta Value	Standardised Beta Value	*t*	*p*	VIF
(Constant)	1.19		3.38	0.000	
Purchase preference					
Flavour	0.05	0.03	1.11	0.265	1.69
Certification marks	−0.06	−0.04	−0.99	0.323	1.57
Added feature	−0.02	−0.03	−0.96	0.336	1.31
Perceived value					
Functional value	0.41	0.43 ***	8.82	0.000	2.28
Social value	−0.05	−0.04	−1.11	0.268	2.04
Conditional value	0.13	0.08 **	2.78	0.006	1.66
Emotional value	−0.22	−0.20 ***	−6.75	0.000	1.62
Marketing					
Promotion marketing	0.71	0.06	1.81	0.070	2.05
Convenience marketing	0.08	0.06	1.90	0.058	1.74
Product marketing	0.09	0.06 *	2.09	0.037	1.51
Price marketing	0.02	0.02	0.82	0.410	1.18
Purchase frequency	0.23	0.24 ***	9.61	0.000	1.12
Model summary	*R* ^2^	0.43			
	*F*	65.20 ***			
	*p*	0.000			

Note: * *p* < 0.05, ** *p* < 0.01, *** *p* < 0.001.

**Table 4:** Regression analysis of WTP (*n* = 1042).

Variable	WTP				
	Nonstandardised Beta Value	Standardised Beta Value	*t*	*p*	VIF
(Constant)	2.56		5.90	0.000	
Purchase preference					
Flavour	0.04	0.03	0.68	0.496	1.68
Certification marks	0.05	0.03	0.72	0.469	1.58
Added feature	−0.03	−0.03	−0.89	0.372	1.29
Perceived value					
Functional value	−0.19	−0.14 ***	−3.36	0.000	2.26
Social value	0.15	0.12 **	3.08	0.002	2.05
Conditional value	0.11	0.07	1.88	0.059	1.65
Emotional value	−0.11	−0.10 ***	−2.72	0.000	1.59
Marketing					
Promotion marketing	0.20	0.17 ***	4.20	0.000	2.06
Convenience marketing	0.08	0.06	1.53	0.126	1.74
Product marketing	−0.22	−0.14 ***	−4.07	0.000	1.51
Price marketing	−0.16	−0.17 ***	−5.64	0.000	1.19
Monthly disposable income	0.16	0.17 ***	6.00	0.000	1.04
Model summary	*R* ^2^	0.19			
	*F*	20.10 ***			
	*p*	0.000			

Note: ** *p* < 0.01, *** *p* < 0.001.

**Table 5:** Independent samples *t* test for gender (*n* = 1042).

Variable	Men (*n* = 497)		Women (*n* = 545)		*t*	Levene Statistic	Degree of Freedom (df)
	*M*	*SD*	*M*	*SD*			
Purchase intention	4.47	0.92	4.26	0.99	3.63 ***	0.63	1040
WTP	2.73	1.06	2.56	0.90	2.76 **	19.12	975.22

Note: ** *p* < 0.01, *** *p* < 0.001.

**Table 6:** One-way ANOVA for educational level (*n* = 1042).

Variable	Senior High School or Vocational School or Less a (*n* = 162)		University or Junior College b (*n* = 546)		Graduate Degree c (*n* = 334)		*F*	Levene Statistic	*df*	Scheffé Test
	*M*	*SD*	*M*	*SD*	*M*	*SD*				
Purchase intention	4.57	0.99	4.27	0.92	4.38	0.95	6.29 **	0.03	2	a > b
WTP	2.34	0.89	2.50	0.86	3.02	1.10	41.46 ***	21.75	2	c > a, b

Note: ** *p* < 0.01, *** *p* < 0.001.

**Table 7:** One-way ANOVA for monthly disposable income level (*n* = 1042).

Variable	≤10,000 a (*n* = 171)		10,001–30,000 b (*n* = 359)		30,001–50,000 c (*n* = 228)		≥50,001 d (*n* = 284)		*F*	Levene Statistic	*df*	Scheffé Test
	*M*	*SD*	*M*	*SD*	*M*	*SD*	*M*	*SD*				
Purchase intention	4.35	1.04	4.32	0.93	4.34	0.94	4.43	0.98	0.80	1.00	3	
WTP	2.42	0.87	2.48	0.81	2.57	0.82	3.05	1.22	24.44 ***	29.74	3	d > a, b, c

Note: *** *p* < 0.001.

**Table 8:** One-way ANOVA for most frequently purchased pork product type (*n* = 1042).

Variable	Frozen a (*n* = 95)		Chilled b (*n* = 345)		Fresh c (*n* = 568)		Processed d (*n* = 34)		*F*	Levene Statistic	*df*	Scheffé Test
	*M*	*SD*	*M*	*SD*	*M*	*SD*	*M*	*SD*				
Purchase intention	4.28	0.94	4.27	0.97	4.44	0.94	4.14	1.18	3.29	1.21	3	
WTP	3.05	1.16	2.80	1.02	2.46	0.87	2.91	1.16	16.20 ***	10.22	3	a, b > c

Note: *** *p* < 0.001.

**Table 9:** One-way ANOVA for purchase frequency (*n* = 1042).

Variable	A (*n* = 10)		B (*n* = 212)		C (*n* = 505)		D (*n* = 137)		E (*n* = 178)		*F*	Levene Statistic	*df*	Scheffé Test
	*M*	*SD*	*M*	*SD*	*M*	*SD*	*M*	*SD*	*M*	*SD*				
Purchase intention	5.00	0.79	4.77	0.86	4.45	0.80	3.14	0.98	3.74	1.15	35.47 ***	5.03	4	a, b, c > d, e
WTP	2.60	1.35	2.58	1.02	2.64	0.99	2.88	1.03	2.56	0.84	2.54 *	1.56	4	

Note 1: * *p* < 0.05, *** *p* < 0.001. Note 2: A = daily, B = once per 2–3 days, C = once per week, D = once per month, E = rarely.

## Data Availability

Data availability upon requested.

## Appendix A

**Table A1:** Variables and Items.

Variables/Items		References
Purchase preference: Consumers’ preference in purchase,		[3,5,10,33,34,60,61]
1.	Juicy pork.	
2.	Tender pork.	
3.	Sweet and savoury pork.	
4.	Pork with a pink surface.	
5.	Pork with an elastic or firm texture.	
6.	Specific cuts (e.g., tenderloin, belly, and chop).	
7.	Pork with high marbling.	
8.	Pork without a raw smell.	
9.	Pork with a Traceable Agricultural Products certificate.	
10.	Pork with a certificate of origin.	
11.	Pork with hygiene, safety, and antibiotic residue-free certification.	
12.	Pork from farms that comply with relevant animal welfare regulations.	
13.	Pork of top quality (e.g., from pigs without swine flu).	
14.	Pork from domestic farms.	
15.	Imported pork.	
16.	Processed and flavoured pork.	
Perceived value: Consumers’ overall evaluation of a product or service		[12,13,23,37,40,41,45]
1.	I feel happy when I see a table full of pork dishes that I can indulge in.	
2.	I feel satisfied when buying a large quantity of pork for cooking.	
3.	I feel full more easily when eating pork.	
4.	Pig organs (e.g., liver and kidney) contain rich vitamins and serve as excellent tonics.	
5.	Pork is more affordable than other husbandry products.	
6.	Pork is rich in iron.	
7.	Pork is rich in protein.	
8.	Pork vendors in traditional markets are more friendly.	
9.	Pork can serve as an alternative staple food.	
10.	I want to try imported pork of special varieties (e.g., Iberian pig).	
11.	Buying and eating imported pork makes my life more enjoyable.	
12.	Pork from pigs fed with special feeds has special flavour.	
13.	I feel a sense of superiority when buying expensive pork from famous brands.	
14.	Group buying of branded pork through friends in online communities is exciting and increases community identity.	
15.	Pork from specialist shops or department stores that are clean and well-lit is classier.	
16.	Vendors provide information on the texture and characteristics of each cut.	
17.	Cooking methods or recipes are available for different types of pork.	
18.	Purchasing pork from domestic farms is an act supporting the local industry.	
19.	Directly purchasing pork from farms is beneficial to farmers.	
20.	Matsusaka pork is rare and expensive because for each pig only two cuts can be obtained, from the jowl and the cheek.	
21.	Pig farming is associated with a dirty environment.	
22.	Eating pork gives a sense of guilt for the slaughter of pigs.	
Marketing mix: The effective integration of marketing strategies and activities during product sales		[8,41,48,55,57,59,60,62]
1.	Purchase branded pork recommended by celebrities, Internet celebrities, or bloggers.	
2.	Purchase branded pork recommended by connoisseurs or restaurant chefs.	
3.	Purchase branded pork that appears on television news reports.	
4.	Purchase branded pork promoted in person by vendors.	
5.	Purchase pork from e-commerce platforms.	
6.	Purchase expensive pork.	
7.	Purchase pork from department stores or brand retailers	
8.	Purchase pork from famous brands.	
9.	Purchase pork that has been sliced and packaged for easy storage	
10.	Purchase pork in vacuum packages.	
11.	Purchase pork from chain supermarkets or retailers	
12.	Purchase pork from meat specialty shops.	
13.	Purchase pork from nearby convenience stores.	
14.	Purchase branded pork recommended by governmental or agricultural authorities.	
15.	Purchase pork at a discounted price.	
16.	Purchase pork at a low price.	
17.	Purchase fresh or chilled pork.	
18.	Purchase pork from vendors in traditional markets.	
19.	Purchase pork with certification marks.	
Purchase intention: A tendency to purchase a product or service		[8,22,23]
1.	Consider buying pork first before other food items.	
2.	Prioritise buying pork over other food items.	
3.	Purchase pork frequently.	
WTP: The maximum price that a consumer is willing to pay for a product		[9,26,27]
How much am I willing to buy imported pork with quality assurance?		
